# Two-Dimensional Plasma Soft X-ray Radiation Imaging System: Optimization of Amplification Stage Based on Gas Electron Multiplier Technology

**DOI:** 10.3390/s24165113

**Published:** 2024-08-07

**Authors:** Karol Malinowski, Maryna Chernyshova, Sławomir Jabłoński, Tomasz Czarski, Andrzej Wojeński, Grzegorz Kasprowicz

**Affiliations:** 1 Institute of Plasma Physics and Laser Microfusion, Hery 23, 01-497 Warsaw, Poland; karol.malinowski@ifpilm.pl (K.M.); slawomir.jablonski@ifpilm.pl (S.J.); tomasz.czarski@ifpilm.pl (T.C.); 2 Institute of Electronic Systems, Warsaw University of Technology, Nowowiejska 15/19, 00-665 Warsaw, Poland; andrzej.wojenski@pw.edu.pl (A.W.); grzegorz.kasprowicz@pw.edu.pl (G.K.)

**Keywords:** gaseous ionization detector, gas electron multiplier (GEM), X-ray (SXR) radiation imaging, tokamak plasma diagnostics

## Abstract

The objective of the proposed research is to develop plasma soft X-ray (SXR) radiation imaging that includes spectral information in addition to standard SXR tomography for the purpose of studying, for example, tungsten transport and its interplay with magnetohydrodynamics (MHD) in tokamak plasmas in an ITER-relevant approach. The SXR radiation provides valuable information about both aspects, particularly when measured with high spatial and temporal resolution and when tomographic reconstructions are performed. The spectral data will facilitate the tracking of both light and high-Z impurities. This approach is pertinent to both the advancement of a detailed understanding of physics and the real-time control of plasma, thereby preventing radiative collapses. The significance of this development lies in its ability to provide three-dimensional plasma tomography, a capability that extends beyond the scope of conventional tomography. The utilization of two-dimensional imaging capabilities inherent to Gas Electron Multiplier (GEM) detectors in a toroidal view, in conjunction with the conventional poloidal tomography, allows for the acquisition of three-dimensional information, which should facilitate the study of, for instance, the interplay between impurities and MHD activities. Furthermore, this provides a valuable opportunity to investigate the azimuthal asymmetry of tokamak plasmas, a topic that has rarely been researched. The insights gained from this research could prove invaluable in understanding other toroidal magnetically confined plasmas, such as stellarators, where comprehensive three-dimensional measurements are essential. To illustrate, by attempting to gain access to anisotropic radiation triggered by magnetic reconnection or massive gas injections, such diagnostics will provide the community with enhanced experimental tools to understand runaway electrons (energy distribution and spatial localization) and magnetic reconnection (spatial localization, speed…). This work forms part of the optimization studies of a detecting unit proposed for use in such a diagnostic system, based on GEM technology. The detector is currently under development with the objective of achieving the best spatial resolution feasible with this technology (down to approximately 100 µm). The diagnostic design focuses on the monitoring of photons within the 2–15 keV range. The findings of the optimization studies conducted on the amplification stage of the detector, particularly with regard to the geometrical configuration of the GEM foils, are presented herein. The impact of hole shape and spacing in the amplifying foils on the detector parameters, including the spatial size of the avalanches and the electron gain/multiplication, has been subjected to comprehensive numerical analysis through the utilization of Degrad (v. 3.13) and Garfield++ (v. bd8abc76) software. The results obtained led to the identification of two configurations as the most optimal geometrical configurations of the amplifying foil for the three-foil GEM system for the designed detector. The first configuration comprises cylindrical holes with a diameter of 70 μm, while the second configuration comprises biconical holes with diameters of 70/50/70 μm. Both configurations had a hole spacing of 120 μm.

## 1. Introduction

Research on tokamak-based fusion plasma systems has the potential to revolutionize the production of clean and affordable electricity. A crucial aspect of this research is the interaction between the plasma and the tokamak chamber wall, which results in the formation of impurities in the plasma, including carbon and tungsten ions from the wall materials [1]. Systems are required to detect heavier element ions in the plasma as their presence can cause a loss of power by the fusion plasma [2]. In addition, uncontrolled runaway electron generation is a concern in fusion plasma research because of its potential for breaking the plasma core or damaging the machine [3]. When interacting with ions or an external magnetic field, electrons can also generate X-rays [4]. Therefore, X-ray-detector-based diagnostics are necessary to obtain valuable information about both impurity ions and electrons in the plasma, providing data on their spatial and energy distribution. 

Soft X-ray (SXR) emission from the tokamak plasma has a variety of potential applications. SXR-based diagnostics have been widely used for monitoring and analyzing impurity concentrations, detecting core magnetohydrodynamics (MHD) modes, identifying core islands, and more [5]. It is important to acknowledge that the current tomographic diagnostic techniques based on SXR have inherent limitations in terms of spatial resolution. The utilization of tangential imaging facilitates enhanced spatial resolution; however, the interpretation of the resulting images necessitates the employment of sophisticated inversion techniques, predominantly due to the integration of three-dimensional line-of-sight data [6]. Nevertheless, it is thought that 2D SXR plasma photon counting imaging [7] has considerable potential for use in magnetic fusion experiments. 

The proposed research extends beyond the conventional scope of SXR tomography by offering not only spectral resolution to track both light and high-Z impurities, but also the capacity for three-dimensional tomography of plasma. This represents a significant advancement over the capabilities of conventional tomography. This offers an opportunity to detect and study azimuthal asymmetry (that has been hardly explored so far) due to plasma non-axisymmetry. As plasma shape control in DEMO cannot be assured by magnetic measurements due to screening effects, the development of alternative techniques, such as SXR diagnostics, is of great importance. Such imaging diagnostics can be beneficial for the identification of magnetic axis (a similar project was already initiated [8]) due to the equilibrium uncertainty. It could provide a future technology to monitor the shape in some devices where magnetics measurements will be ineffective.

The intrinsic characteristics of GEM detectors render them particularly well-suited to the challenging environment anticipated for the proposed tool. Thus far, gas detectors appear to be one of the most reliable and least susceptible to degradation over time, in contrast to semiconductor or MCP detectors, which have been observed to lose sensitivity several times over the course of a few years. Furthermore, gas detectors are less susceptible to low-energy quanta, such as stray visible or infrared radiation, in comparison to, for instance, CCDs. The preliminary studies [9] demonstrate that GEM detectors are less susceptible to damage from neutrons than, for example, semiconductor-type detectors, and the recorded neutron-related pulses can be distinguished from those associated with SXR through pulse height analysis [10].

In addition to gathering qualitatively new information, the proposed development offers the capability of photon-to-signal conversion efficiency and excellent spatial resolution [11]. The latter is dependent on the port geometry and alignment outside the vessel. The viewing area can be selected by moving the entire setup, i.e., zooming in and out. It is anticipated that a resolution of approximately 0.1 mm (at the detector readout plane) will be achieved. This will provide a unique opportunity to resolve small-scale phenomena within the plasma cross-section. Spectral information (approximately 20%) is provided by itself with no added discrimination tools or filters. Although the 2D imaging system development was already launched, it did not meet the needs of plasma physics (not capable of recording high photon fluxes or having poor spatial resolution). The proposed advanced imaging diagnostics are devoted to overcoming these inabilities.

Convenient silicon detectors [12,13] are typically employed in all major tokamaks for SXR tomography. However, in burning plasma experiments, during the D-T phase of ITER operation, it is unlikely that line-of-sight detection of SXRs based on semiconductor technology will be sufficiently hardened against radiation. The solution might be to position those in well-shielded locations [14], which means no direct imaging will be provided by such systems. However, as observed on the tokamak TFTR during D-T operation [15,16], even with perfect shielding against indirect neutrons, silicon-based detectors would age rapidly in such an environment since neutron irradiation reduces the strength of structural materials. This necessitates frequent replacement of detectors in the presence of burning plasma.

In turn, digital X-ray imaging techniques have undergone rapid development and are employed in a variety of detectors, including X-ray image intensifiers, X-ray CCDs, CMOS sensors, semiconductor detectors, and flat-panel detectors for radiographic applications. High spatial resolution and high detection efficiency are among the advantages of these devices together with good stability. However, they have limited active area and low sensitivity to low-energy X-rays.

In contrast, gaseous detectors, which are the focus of this study, can readily cover extensive active regions. The recently developed Micro-Patterned Gaseous Detectors (MPGDs) exhibit enhanced capabilities in charge amplification and high spatial resolution, which substantiates their suitability for detecting low-energy radiation, including β-rays and low-energy X-rays. This has been demonstrated with the integration of appropriate charge-readout electronics.

Scintillator-based diagnostics as an imaging method has also been developed for applications within plasma fusion [17,18]. Although, these detectors are resilient to neutrons/γ-rays, their time decay is at least 3–4 times worse than the signal duration for the proposed GEM detector, and the spatial resolution of the system is constrained by the physical dimensions of a plastic fiber within a bundle (100 × 100) [17] rather than the resolution of the video camera. Nevertheless, they are still based on semiconductor technology, subject to neutron-induced damage (e.g., [19,20]). 

The proposed SXR imaging diagnostics, based on GEM detectors [21], ensures that each photon is detected individually, and its energy is estimated accordingly. The system offers three key advantages. Firstly, it improves the physical parameters on which imaging quality is based. Secondly, it provides genuinely “digital” detection, which allows easy data post-processing. This may involve, for example, combining adjacent pixels or carrying out time integration, resulting in an increase in the total signal or SNR or contrast. Thirdly, it enables energy discrimination in bands of the X-ray photons, providing a “color X-ray radiography and/or tomography”.

The targeted range of photon energy detection is between 2−15 keV. It is anticipated that the detector window and designed readout electrode will have a surface area of approximately 10 × 10 cm^2^. The readout board is a two-dimensional matrix comprising approximately 33,000 hexagonal pixels, each with a side length of 0.35 mm [22]. This enables excellent spatial resolution for performing 3D tomography of the plasma, thereby providing an opportunity to study the toroidal anisotropy of the plasma.

**Figure 1:**
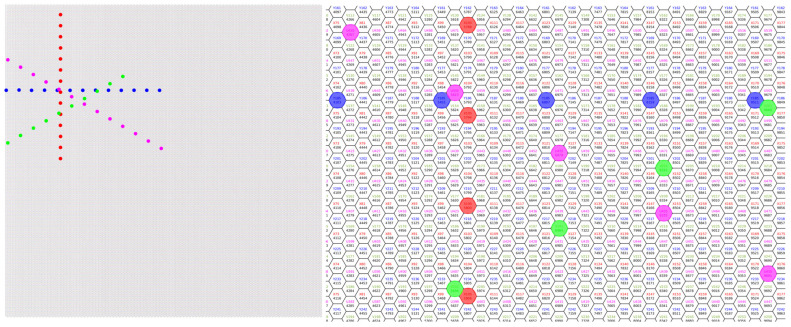
View of the matrix with examples of pixel connections. The identical color of the circle indicates the connected pixels. This type of merging results in an approximately 11-fold reduction in actual signal channels. However, it does necessitate the simultaneous recording of the signal generated by a single photon absorption on two adjacent pixels. Furthermore, a more uniform loading of the signal channels is ensured when the detector area is unevenly irradiated by photons. The left-hand image depicts the entire matrix, while the right-hand image is a more detailed section. The colors of the numbers relate to the direction of merging, with the same numbers denoting merged pixels.

In order to maintain the effective spatial resolution of the detector at the level of 33,000 pixels while reducing the number of electronics channels, a special pixel connection system was implemented. Figure 1 provides a general overview of the matrix, with illustrative examples of pixel combinations. The pixels are connected in four directions, in groups of 12–14 pixels, and occupy the area from the edge to the center of the matrix (to the directional symmetry axis of the connected pixels). The same methodology as that employed in references [23,24] was utilized. The system reduced the number of actual signal channels to approximately 3000 by requiring that the signal generated by a single X-ray photon be recorded by at least two adjacent pixels in a time-coincident manner. This solution permits a more uniform loading of signal channels, even when there are considerable spatial variations in the intensity of incident photons on the detector window. 

In order to determine the optimal operating parameters of the constructed detector, in particular the maximum spatial size of the avalanches with maximum electronic gain, comprehensive simulations were conducted. The impact of the geometric parameters of the GEM foil, specifically the stage of detector amplification, on the electron avalanche formation and registration process, including the shape of the holes and their spacing, was the primary focus of the study.

## 2. Methodology

**Figure 2:**
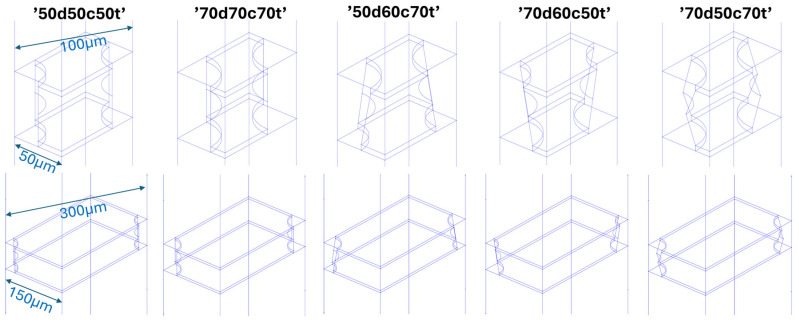
Cell models of the considered GEM foil cases: hole spacing ranging from 100 μm (**top**) to 300 μm (**bottom**) in 10 μm increments. The hole geometries, from left to right, are as follows: cylindrical Ø50 μm, cylindrical Ø70 μm, cup-shaped Ø70/50 μm, inverted cup-shaped Ø50/70 μm, and biconical Ø70/50/70 μm. The foil thickness of Kapton is 50 μm, with a 5 μm copper layer coating both sides.

The most commonly utilized GEM foils are typically manufactured with double-cone holes with outer and inner diameters of 70 μm and 50 μm, respectively, and holes spaced at 140 μm intervals. However, it is possible to alter the technological process to produce films with different geometrical parameters [25]. In the frame of this research, the aim was to develop a suitable geometry for a GEM detector amplification stage for use in plasma radiation studies. Consequently, in order to identify the foil geometry that yields the greatest spatial extent of avalanches with the highest electron gain, the impact of two parameters—the shape of the holes and their spacing—was investigated in the simulation process. The study examined five different hole shapes, as illustrated in Figure 2. The shapes examined included a cylindrical shape with a diameter of 50 μm, a cylindrical shape with a diameter of 70 μm, a cup shape with diameters of 70/50 μm, an inverted cup shape with diameters of 50/70 μm, and a biconical shape with diameters of 70/50/70 μm. The designations, such as ‘50d60c70t’, indicate the diameter, in micrometers, of the hole on the drift side, in the center, and on the transfer side, respectively. The spacing between the holes was increased from 100 to 300 μm in increments of 10 μm. This resulted in 105 distinct sets of GEM foil geometry parameters, which were investigated for their potential impact on detector operation. The detector simulation retained constant values for several parameters, including a gas mixture of Ar/CO_2_ in a 70/30 ratio, GEM Cu/Kapton/Cu foil thicknesses of 5/50/5 μm, and gas space thicknesses for D/T1/T2/I (Drift/Transfer1/Transfer2/Induction) of 5/2/2/2 mm. The electric field values for D, T1, T2, and I were maintained at a constant 3 kV/cm throughout the simulations. A high voltage of 365 V was applied to a GEM foil, resulting in an electric field of 73 kV/cm.

The simulations examined the parameters of the electron avalanches generated within a GEM-type detector as a result of interactions with 6 keV photons (such as those emitted by the ^55^Fe isotope laboratory source) across all considered GEM foil geometry configurations. The calculations were performed using Garfield++ software [26], which is a computer program designed for the construction of detailed simulations of multiple aspects of drift chambers with gaseous media. For a given electric field, the program allows the simulation of drift and the entire process of creating an avalanche of electrons in the gas. In order to calculate the requisite electron transport coefficients in the gas, Garfield++ employs another program, Magboltz [27], which is used to solve the Boltzmann transport equations for electrons in gas mixtures under the influence of electric and magnetic fields. As Garfield++ requires the use of external software for the generation of more complex electric field geometries, a set of two programs, Gmsh [28] and Elmer [29,30], were employed for the calculation of the electric field distribution in the GEM detector for all cases under investigation. Gmsh is a finite-element mesh (FEM) generator that is employed for the purpose of defining a given geometry and subsequently splitting it into numerous discrete elements. This enables the finite element method to be utilized for the calculation of electrostatic fields. Elmer is a finite element analysis program that is employed for the calculation of the electric field throughout space in a device geometry utilizing a mesh that has been previously generated by Gmsh. The three-dimensional map of the electric field, prepared in accordance with the aforementioned methodology, was subsequently employed in the calculations conducted with the Garfield++ software. For each configuration, 10^4^ avalanches were tallied. A calculation performed on a 56-threaded workstation required a significant amount of time, spanning several weeks, to complete.

Considering the anticipated significance of the spatial distribution of primary electrons initiating the electron avalanche (their relative position to each other and their cluster position in the drift volume relative to the gem foil) originating from a single X-ray photon on the simulation results, the Degrad program [31] was employed. Degrad is capable of calculating the electron cluster size distribution and the distribution of primary electrons in gas mixtures. This is achieved using an accurate Auger cascade model for the interaction of photons and particles with gas mixtures in electric and magnetic fields. The use of the Degrad software allowed for a more accurate representation of the spatial positions of the primary electron clusters, taking into account the specific characteristics of the gas mixture and the energy of the X-ray photons. This information was crucial for the accurate simulation of the electron avalanche and the subsequent gas amplification process in the GEM film.

**Figure 3:**
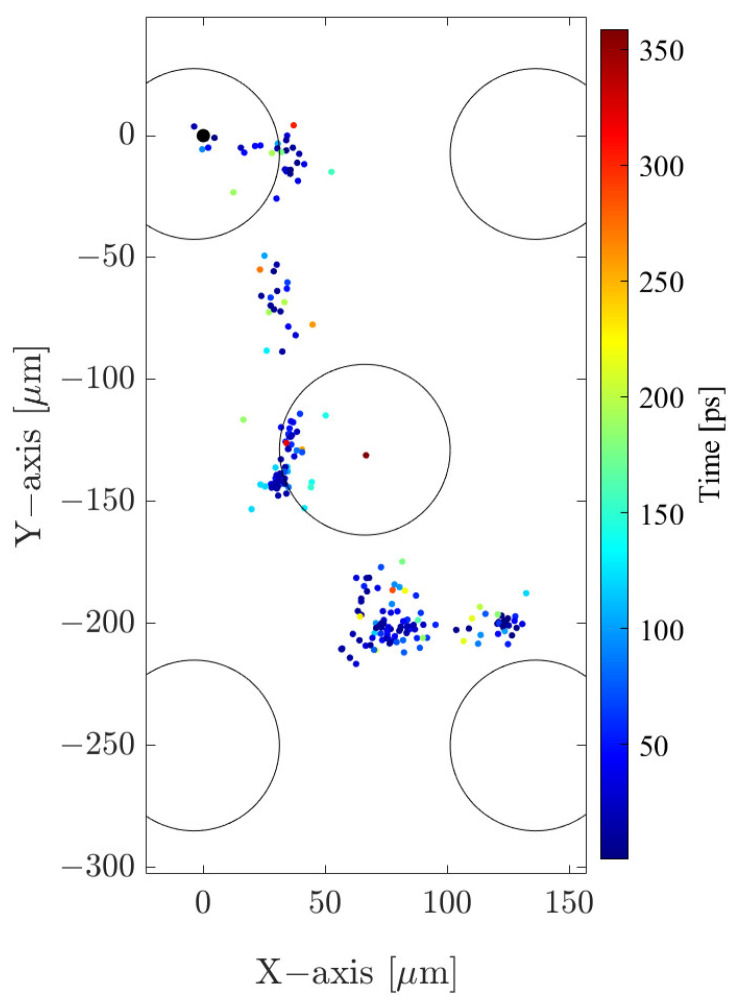
An illustrative example of the results obtained from Degrad. The figure illustrates the spatial distribution of primary electrons (in the XY plane) that have been thermalized to 2 eV. This distribution is a consequence of the absorption of a 6 keV photon at the point (0, 0) in a 70/30 Ar/CO_2_ gas mixture under an electric field of Ez = 3 kV/cm. The black circles represent the size of holes in the GEM foil with Ø70 μm and 140 μm pitch, included for scale reference.

The utilization of this program resulted in the preparation of a database comprising the coordinates of primary electron positions within the drift region, originating from 10^4^ photons with an assumed energy of 6 keV. The database served as the set of input parameters for simulating avalanches in Garfield++ and remained consistent across all examined GEM foil geometry configurations. Figure 3 presents an example, obtained with the Degrad program, of the spatial distribution of primary electrons formed via the photoelectric absorption of a single photon in the drift region.

By simulating the avalanche in Garfield++ using the prepared database of primary electron positions, it was possible to study the effect of different GEM film geometries on the gas amplification factor and the overall detector performance. This approach provided valuable insights into the factors influencing the efficiency and sensitivity of the detector system, which can be used to optimize its design for specific applications.

## 3. Results Discussion

**Figure 4:**
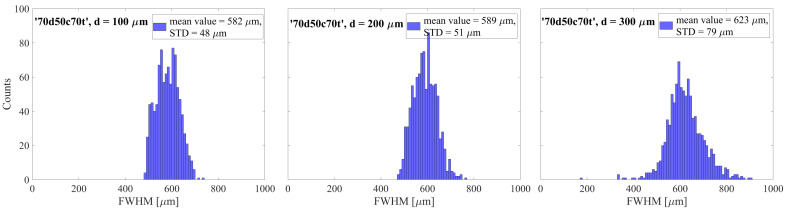
FWHM distributions of avalanches on the readout electrode for the ‘70d50c70t’ case with a hole spacing of 100, 200, and 300 µm. The FWHM values were determined by fitting single avalanche distributions with the Voigt distribution.

The initial parameter examined based on the simulation results was the electron spot size on the readout electrode. The cross-sectional profiles along the X and Y axes of the spatial distributions from individual avalanches were fitted with Voigt distributions. The full width at half maximum (FWHM) value, which serves as a measure of the spatial extent of a given avalanche, was obtained by averaging over both dimensions. Figure 4 illustrates the distributions of FWHM values obtained through this methodology for 10^4^ avalanches in the case with hole shape ‘70d50c70t’ (μm) and hole spacing of 100, 200, and 300 μm. 

**Figure 5:**
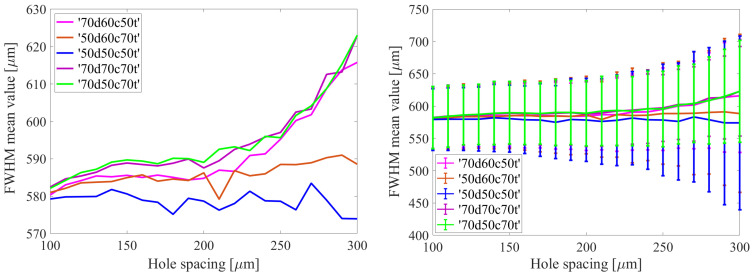
Distribution of FWHM values of electron avalanches on the readout electrode for different GEM foil configurations, including variations in hole shape and spacing. The right side of the figure displays the same distribution with the standard deviation indicated.

Subsequently, the mean values and standard deviations were calculated for these distributions as measures of the dispersion of the measured spot width values from the mean. The results, shown in Figure 5, indicate that the mean FWHM values and their standard deviations do not exhibit a significant change for hole spacings ranging from 100 to 200 μm. However, beyond this range, there is an observable increase in both the mean FWHM values and standard deviation. In general, the mean FWHM values fall within the range of 580–620 μm, representing a difference of approximately 7%. However, there is an exception observed in the cases of ‘50d60c70t’ and ‘50d50c50t’, where the FWHM value remains practically constant but the scatter of its value increases approximately twofold for larger hole spacings.

The diameter of the electron spot on the reading electrode, as obtained from the simulation, was compared with the value obtained from experimental data. In [32], a statistical method was employed to obtain, from experimental data, the dependence of the electron spot diameter on the energy of the X-ray photon, which serves as the source of the avalanche. The value obtained in the aforementioned work, approximately 600 μm for 6 keV, was compared with the value obtained from simulations in the present work, approximately 590 μm for the analogous case (‘70d50c70’, 140 μm hole spacing). When accounting for discrepancies such as the voltage applied to the GEM foil and the different methodologies for defining the diameter, the avalanche sizes obtained from the simulations presented here and in previous experiments [32] demonstrate a high degree of agreement.

The analysis of the simulation results revealed that for X-rays with an energy of 6 keV, the width of the electron spot on the readout electrode, measured at 10% of the maximum value, is approximately 1 mm, with variations between configurations similar to those observed for the FWHM values. Given the dimensions of the pixel, a hexagon with a side length of 0.35 mm, it can be concluded that even for lower energies, the signal from a single electron avalanche should be registered by at least two pixels.

The electron gain was determined for each avalanche by calculating the ratio of the number of electrons that reached the readout electrode to the number of primary electrons. Subsequently, the mean gain value and its standard deviation were calculated. In order to facilitate the evaluation of the results, the gain resolution parameter, defined as the ratio of the standard deviation to the mean value (gain std/gain), was introduced. This parameter enables the determination of the width of the electron gain distribution, which is of crucial importance for the performance of the detector and translates into the measured resolution of charge (energy) distributions. The ability to distinguish peaks/contributions in the spectrum from two different discrete X-ray energies improves as the value of this parameter decreases.

**Figure 6:**
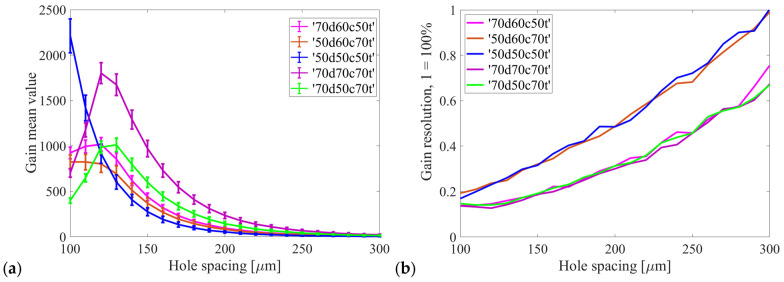
(**a**) The mean electron gain of the GEM detector calculated for all cases under study. (**b**) The resolution of the gain distributions, which is defined as the width of the distribution divided by its mean value.

Figure 6 shows the obtained results, indicating that the maximum gain values for the studied configurations are achieved with hole spacings within the range of 120–130 µm, with the exception of the ‘50d50c50t’ configuration, which reaches its maximum gain with a spacing of less than 100 µm. The ‘70d70c70t’ cylindrical configuration yields the largest gain values overall. The optimal hole spacing for the gain resolution is 100–130 µm. In contrast, the ‘50d60c70t’ and ‘50d50c50t’ configurations exhibit the least favorable values. In relation to Figure 6b, it should be noted that the exemplar actual energy resolution value of the GEM detector is approximately 22% [33], whereas the corresponding value derived from simulations is approximately 17% in the case of the ‘70d50c70t’ configuration with 140 µm hole spacing. Nevertheless, the shape of the presented curve indicates that reducing the hole spacing to 120 µm should improve the actual energy resolution of the detector.

**Figure 7:**
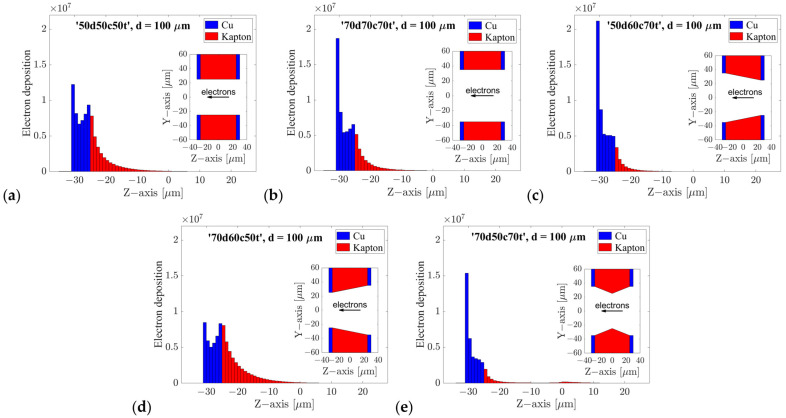
(**a**–**e**) The spatial distribution of electrons that terminate their trajectories on the walls of the hole for all the examined configurations. The charge deposited by 10^4^ electron avalanches on the Kapton (red) is presented with the respective values being 30 Me, 17 Me, 9.4 Me, 40 Me, and 7.2 Me for a given configuration. The fraction relative to the total number of electrons in the hole is 0.27, 0.20, 0.12, 0.40, and 0.12, respectively. The center of the hole is located at the z = 0 point. The section ranging from −25 to 25 µm represents the Kapton component, whereas the sections ranging from −30 to −25 µm and 25 to 30 µm represent the copper component, which comprises the electrodes on the foil.

In the process of selecting the most optimal configurations, the effect of electron deposition on the Kapton part of the holes in the GEM foils was investigated as an additional parameter. This parameter is of great importance, as electron deposition alters the electric field distribution in the hole, which in turn leads to instability in detector performance due to changes in gain value. Figure 7 presents the quantitative distribution of charge deposition on the hole walls for the five tested hole shapes with 100 µm spacing.

**Figure 8:**
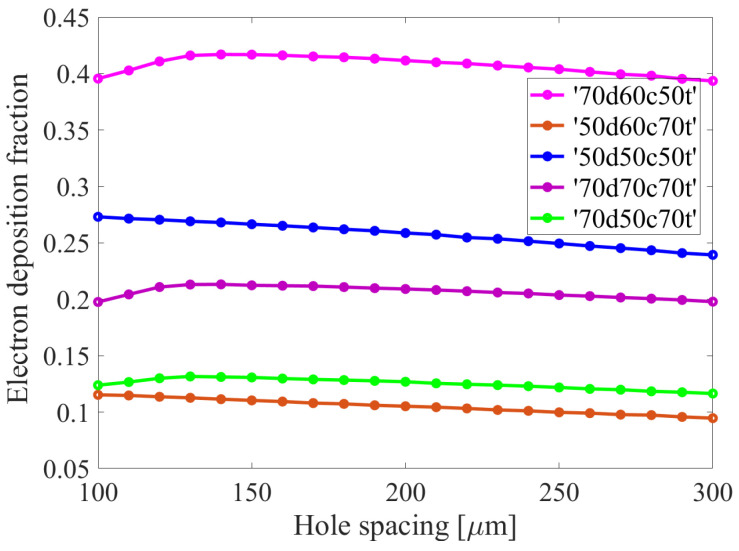
The fraction of electron deposition on the GEM film for all cases under investigation. This parameter is defined as the ratio of the number of electrons deposited on the Kapton surface to the total number of electrons passing through the hole, including both primary and secondary electrons.

The results presented in Figure 7 correspond to the absolute value of the charge (in electrons) deposited on Kapton surface (red) in the holes of the third GEM foil (third multiplication stage) as a consequence of the propagation of avalanches initiated by 10^4^ X-ray photons with the specified parameters. Furthermore, Figure 8 illustrates the fractional value in relation to the total number of electrons propagating and arising in the holes. When comparing the configurations under investigation, this fractional value is the most meaningful. As indicated by the obtained data, the fractions of deposited electrons are smallest in the ‘50d60c70t’ and ‘70d50c70t’ configurations.

## 4. Summary

This study is focused on the development of SXR imaging diagnostics, based on the principles of GEM detectors, with the objective of making them applicable to current and future plasma fusion devices. The research is aimed at optimizing the internal structure of the detector for the effective detection of electron signals produced by a single photon. In order to achieve excellent spatial resolution, a large number of readout pixels is required. Therefore, the data acquisition system’s complexity had to be optimized by reducing the number of independent channels. An advanced readout electrode comprising a specific combination of pixels was developed for this application, with the objective of efficiently recording the generated electron avalanches. This required a specific spatial distribution of the generated charge within the detector gas volume.

In light of the aforementioned considerations, the calculations included five hole geometries with cylindrical, biconical, and cup-shaped shapes, whose typical dimensions considered were 70 and 50 µm, with a range of hole spacing from 100 µm to 300 µm. The results demonstrated that modifying the film geometry parameters had a relatively minor impact of approximately 7% on the size (FWHM) of the avalanche spot on the readout electrode. However, the statistical spread of the spot size increased significantly with increasing hole spacing. The results indicated that as the hole spacing increased, the detector gain decreased and the resolution of the gain distributions deteriorated. This would lead to a decrease in the charge/energy resolution of the experimental GEM detector distributions.

The results obtained were used to propose the most optimal geometrical configuration for the three-foil GEM system for the designed detector. The ‘70d70c70t’ GEM foil geometry configuration yielded the most favorable gain values and was identified as the optimal configuration for the designed detector. The ‘70d50c70t’ configuration was determined to be the optimal configuration for the electron deposition fraction parameter and was thus selected as the second-best option. A hole spacing of 120 μm was identified as the most suitable.

## Data Availability

The original contributions presented in the study are included in the article, further inquiries can be directed to the corresponding author.
